# (5*Z*,7*Z*,9*Z*)-5,10-Di­bromo­benzo[8]annulene

**DOI:** 10.1107/S1600536813027797

**Published:** 2013-10-16

**Authors:** Christopher O. Bender, René T. Boeré

**Affiliations:** a Department of Chemistry and Biochemistry, University of Lethbridge, Lethbridge, AB, T1K3M4, Canada

## Abstract

In the structure of the title compound, C_12_H_8_Br_2_, the two bromine substituents are oriented *exo* to the boat-shaped cyclo­octa­tetra­ene at the two ring sites that are β to the ring fusion positions. The average Br—C bond distance is 1.919 (2) Å, the average distance for C=C double bonds that are Br substituted is 1.328 (2) Å, while the other two double-bond distances are 1.327 (2) and 1.398 (2) Å for the non-fused and fused bonds, respectively. Each type of ring inter­atomic distance is within s.u. of the average values for the four known structures, including the title compound, of benzo-fused cyclo­ocata­tetra­enes that are not coordinated to a metal atom. The crystal structure features short Br⋯Br [3.6620 (3) Å] and C⋯H [2.834 (2) and 2.841 (2) Å] contacts.

## Related literature

For general background to photochemical conversions of benzo­cyclo­octa­tetra­enes, see: Bender *et al.* (1982 ▶, 1986 ▶, 1988 ▶, 1991 ▶). For details of the synthesis, see: Barton *et al.* (1964 ▶). For related structures, see: Bohshar *et al.* (1984 ▶); Çelik *et al.* (2002 ▶); Jones *et al.* (1994 ▶), Kidokoro *et al.* (1983 ▶); Li *et al.* (1983 ▶). For a description of the Cambridge Structural Database, see: Allen (2002 ▶). For the *PLATON* suite of crystallographic software, see: Spek (2009 ▶).
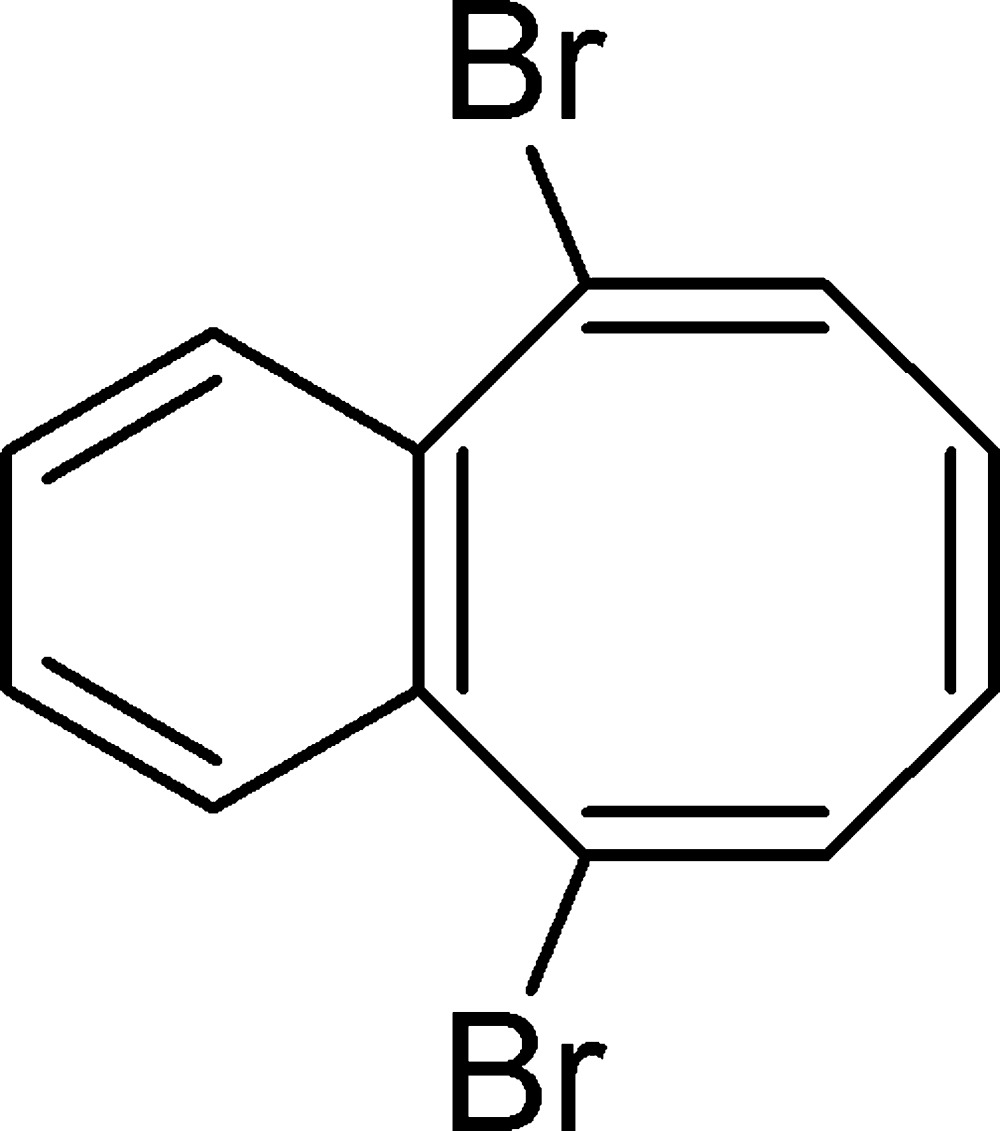


## Experimental

### 

#### Crystal data

C_12_H_8_Br_2_
*M* *_r_* = 312.00
Monoclinic, 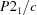

*a* = 8.5289 (5) Å
*b* = 8.3630 (5) Å
*c* = 15.5645 (9) Å
β = 105.2980 (6)°
*V* = 1070.83 (11) Å^3^
*Z* = 4
Mo *K*α radiation
μ = 7.52 mm^−1^
*T* = 173 K
0.16 × 0.09 × 0.08 mm

#### Data collection

Bruker APEXII CCD area-detector diffractometer
Absorption correction: multi-scan (*SADABS*; Bruker, 2008 ▶) *T*_min_ = 0.510, *T*_max_ = 0.746
15053 measured reflections
2426 independent reflections
2217 reflections with *I* > 2σ(*I*)
*R*_int_ = 0.023

#### Refinement

*R*[*F* ^2^ > 2σ(*F* ^2^)] = 0.017
*wR*(*F* ^2^) = 0.039
*S* = 1.04
2426 reflections
127 parameters
H-atom parameters constrained
Δρ_max_ = 0.49 e Å^−3^
Δρ_min_ = −0.46 e Å^−3^

### 

Data collection: *APEX2* (Bruker, 2008 ▶); cell refinement: *SAINT-Plus* (Bruker, 2008 ▶); data reduction: *SAINT-Plus*; program(s) used to solve structure: *SHELXD* (Sheldrick, 2008 ▶); program(s) used to refine structure: *SHELXL2013* (Sheldrick, 2008 ▶); molecular graphics: *Mercury* (Macrae *et al.*, 2006 ▶); software used to prepare material for publication: *publCIF* (Westrip, 2010 ▶).

## Supplementary Material

Crystal structure: contains datablock(s) general, I. DOI: 10.1107/S1600536813027797/hg5345sup1.cif

Structure factors: contains datablock(s) I. DOI: 10.1107/S1600536813027797/hg5345Isup2.hkl

Supplementary material file. DOI: 10.1107/S1600536813027797/hg5345Isup3.cml

Additional supplementary materials:  crystallographic information; 3D view; checkCIF report

## Figures and Tables

**Fig. 1.**
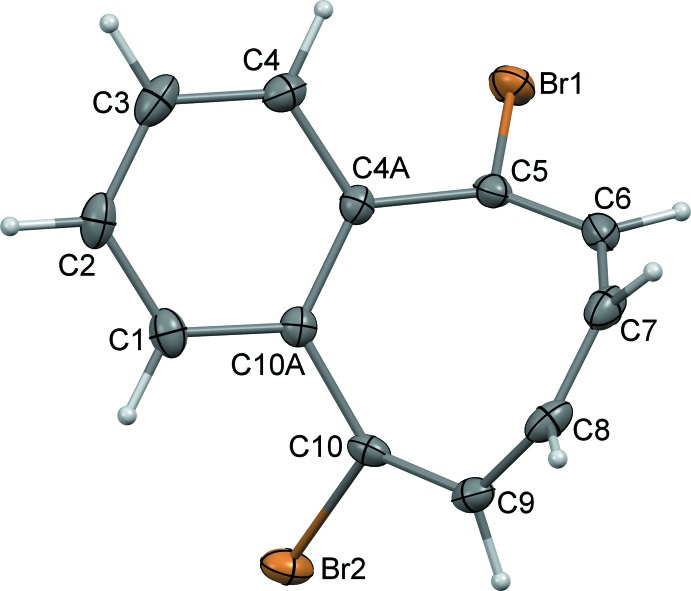
Molecular structure of (I) drawn with displacement elipsoids at the 40% probability level, and also showing the atom numbering scheme.

**Fig. 2.**
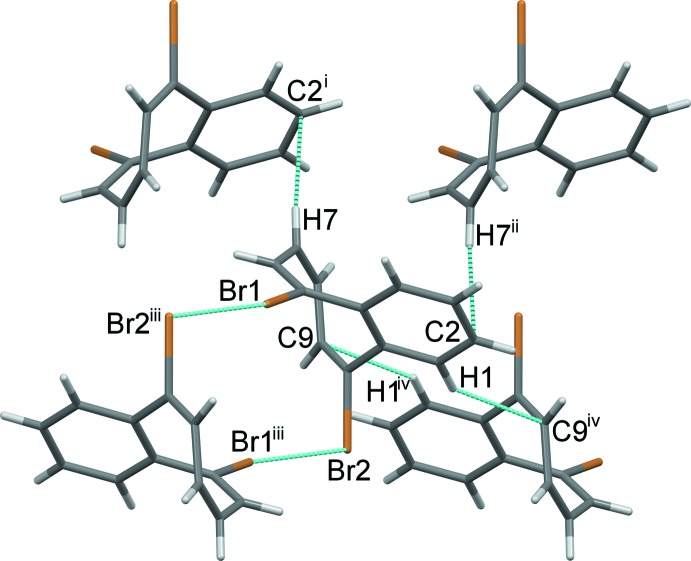
View along the a axis (with b horizontal and c vertical) showing the three unique intermolecular contacts (as dashed-line tubes) that are less than the sums of the v.d. Waals' radii of the interacting atoms. (Symcodes: i, 1-x,-1/2+y,1/2-z; ii, 1-x,1/2+y,1/2-z; iii, -x,1-y,-z; iv, 1-x,-y,-z.)

## supplementary crystallographic information

### 1. Comment

The title compound was prepared as a starting material for synthesis of the corresponding dinitrile derivative, which was of interest in connection with studies of the photochemical conversions of benzocyclooctatetraenes (Bender *et al.*, 1982; 1986; 1988; 1991). In view of the paucity of structures that are crystallographically established for mono-benzofused cyclo-octatetraenes, we decided to undertake a crystallographic study of (I).

Only three prior structures have been reported which contain one benzo-fused cyclooctatetraene ring according to the Cambridge Structural Database (Allen, 2002; WebCSD, August 2013), excluding those with rings coordinated to metals. The parent hydrocarbon, (5*Z*,7*Z*,9*Z*)-benzo[8]annulene (refcode BUYYUP), has been structurally characterized by X-ray crystallography and investigated by semi-empirical quantum mechanical methods (Li *et al.*, 1983). The only halogen-substituted example is (8-chlorobenzocyclooctatetraen-6-yl)-diphenylphosphine-oxide (refcode: CUDYUV) but this ring bears a large Ph_2_P=O substituent; this structure contains two independent molecules in the asymetric unit (Bohshar *et al.*, 1984). In dimethyl 1,4,6,9-tetramethylbenzocyclooctatetraene-5,10-dicarboxylate (refcode: LEZMAE) the two methyl ester substituents are located where the Br atoms are in (I) but this molecule also has four methyl substituents, two attached to the other ends of the same double bonds that the esters are attached to, and two in the 1 and 4 positions of the benzene rings (Jones *et al.*, 1994). Gratifyingly, all the 1,2 interatomic distances in (I) are found to lie within s.u. of the average values from these three comparison structures and (I).

Related benzo-fused cyclooctatrienes which have the same 5,8-dibromo substitution as found in (I) have been structurally characterized. In 5,8,10-tribromo-(8H)-benzocycloocten-7-one (refcode: BOGWAV) the cyclooctatriene ring is distorted by saturation at C8 and a ketone group at C7 (Kidokoro *et al.*, 1983), whilst 5,7,7,8,10-pentabromo-7,8-dihydrobenzocyclooctene (refcode: FABDOC) has C7 with two Br and C8 with H and Br substituents (Çelik *et al.*, 2002). The C5-Br1 and C10-Br2 distances in BOGWAV are 1.8822 (3) and 1.9309 (3) Å and in FABDOC 1.912 (7) and 1.896 (9). Thus the average C-Br bond distance in this set of three related structures is 1.910 (16) Å, with the distances in (I) found within s.u. of this value.

In the crystal structure of (I) molecules are tightly packed. There are three unique short intermolecular contacts (Figure 2), namely C2···H7^i^ = H7···C2^ii^ = 2.834 (2) Å, Br1···Br2^iii^ = 3.6620 (3) Å and C9···H1^iv^ = 2.841 (2) Å (symcodes employed: i, 1-x,-1/2+y,1/2-z; ii, 1-x,1/2+y,1/2-z; iii, -x,1-y,-z; iv, 1-x,-y,-z).

### 2. Experimental

Samples of (I) were prepared from biphenylene via the method of Barton *et al.* (1964). Crystals were obtained from aqueous methanol (m.p. 366–368 K).

### 3. Refinement

All the hydrogen atoms were located on a difference map, but for purposes of refinement are treated as riding on their attached aromatic carbon atoms with C—H = 0.95 Å and *U*_iso_(H) = 1.2*U*_eq_(C).

### Crystal data

**Table:**

C_12_H_8_Br_2_	*D*_x_ = 1.935 Mg m^−^^3^
*M**_r_* = 312.00	Melting point: 366 K
Monoclinic, *P*2_1_/*c*	Mo *K*α radiation, λ = 0.71073 Å
*a* = 8.5289 (5) Å	Cell parameters from 9956 reflections
*b* = 8.3630 (5) Å	θ = 2.5–29.0°
*c* = 15.5645 (9) Å	µ = 7.52 mm^−^^1^
β = 105.2980 (6)°	*T* = 173 K
*V* = 1070.83 (11) Å^3^	Block, colourless
*Z* = 4	0.16 × 0.09 × 0.08 mm
*F*(000) = 600	

### Data collection

**Table:**

Bruker APEXII CCD area-detector diffractometer	2426 independent reflections
Radiation source: fine-focus sealed tube, Bruker D8	2217 reflections with *I* > 2σ(*I*)
Graphite monochromator	*R*_int_ = 0.023
Detector resolution: 66.06 pixels mm^-1^	θ_max_ = 27.4°, θ_min_ = 2.5°
φ and ω scans	*h* = −11→11
Absorption correction: multi-scan (*SADABS*; Bruker, 2008)	*k* = −10→10
*T*_min_ = 0.510, *T*_max_ = 0.746	*l* = −20→20
15053 measured reflections	

### Refinement

**Table:**

Refinement on *F*^2^	Primary atom site location: dual
Least-squares matrix: full	Secondary atom site location: difference Fourier map
*R*[*F*^2^ > 2σ(*F*^2^)] = 0.017	Hydrogen site location: inferred from neighbouring sites
*wR*(*F*^2^) = 0.039	H-atom parameters constrained
*S* = 1.04	*w* = 1/[σ^2^(*F*_o_^2^) + (0.0171*P*)^2^ + 0.5892*P*] where *P* = (*F*_o_^2^ + 2*F*_c_^2^)/3
2426 reflections	(Δ/σ)_max_ = 0.001
127 parameters	Δρ_max_ = 0.49 e Å^−^^3^
0 restraints	Δρ_min_ = −0.46 e Å^−^^3^

### Special details

**Table:**

Geometry. All esds (except the esd in the dihedral angle between two l.s. planes) are estimated using the full covariance matrix. The cell esds are taken into account individually in the estimation of esds in distances, angles and torsion angles; correlations between esds in cell parameters are only used when they are defined by crystal symmetry. An approximate (isotropic) treatment of cell esds is used for estimating esds involving l.s. planes.
Refinement. Structure first solved in *P*2_1_; used the *PLATON* "Addsym" tool to find the true space group and refinement continued in *P*2_1_/c (Spek, 2009).

### Fractional atomic coordinates and isotropic or equivalent isotropic displacement parameters (Å^2^)

**Table:**

	*x*	*y*	*z*	*U*_iso_*/*U*_eq_	
Br1	0.00196 (2)	0.47441 (2)	0.12658 (2)	0.02901 (6)	
Br2	0.33582 (2)	0.24542 (2)	−0.10638 (2)	0.02977 (6)	
C1	0.2640 (2)	−0.0177 (2)	0.03420 (12)	0.0251 (4)	
H1	0.3169	−0.0572	−0.0081	0.030*	
C2	0.1703 (2)	−0.1200 (2)	0.06965 (13)	0.0289 (4)	
H2	0.1598	−0.2291	0.0522	0.035*	
C3	0.0918 (2)	−0.0629 (2)	0.13055 (12)	0.0287 (4)	
H3	0.0285	−0.1332	0.1557	0.034*	
C4	0.1056 (2)	0.0963 (2)	0.15478 (11)	0.0240 (4)	
H4	0.0496	0.1351	0.1958	0.029*	
C4A	0.20053 (19)	0.2013 (2)	0.11986 (10)	0.0187 (3)	
C5	0.2100 (2)	0.3709 (2)	0.14745 (11)	0.0210 (3)	
C6	0.3398 (2)	0.4538 (2)	0.19030 (12)	0.0264 (4)	
H6	0.3235	0.5615	0.2054	0.032*	
C7	0.5062 (2)	0.3917 (2)	0.21606 (12)	0.0282 (4)	
H7	0.5615	0.3944	0.2776	0.034*	
C8	0.5866 (2)	0.3322 (2)	0.16108 (12)	0.0270 (4)	
H8	0.6935	0.2938	0.1867	0.032*	
C9	0.5240 (2)	0.3205 (2)	0.06401 (12)	0.0239 (4)	
H9	0.5851	0.3714	0.0289	0.029*	
C10	0.3892 (2)	0.2449 (2)	0.02106 (11)	0.0206 (3)	
C10A	0.28235 (19)	0.1430 (2)	0.05945 (11)	0.0187 (3)	

### Atomic displacement parameters (Å^2^)

**Table:**

	*U*^11^	*U*^22^	*U*^33^	*U*^12^	*U*^13^	*U*^23^
Br1	0.02787 (10)	0.02768 (10)	0.03510 (11)	0.00464 (7)	0.01468 (8)	−0.00192 (8)
Br2	0.02890 (10)	0.04284 (12)	0.01930 (9)	0.00265 (8)	0.00940 (7)	0.00122 (7)
C1	0.0269 (9)	0.0217 (9)	0.0237 (9)	0.0048 (7)	0.0016 (7)	−0.0011 (7)
C2	0.0294 (9)	0.0177 (9)	0.0326 (10)	−0.0018 (7)	−0.0041 (8)	0.0015 (8)
C3	0.0240 (9)	0.0264 (10)	0.0308 (10)	−0.0070 (7)	−0.0016 (7)	0.0094 (8)
C4	0.0202 (8)	0.0286 (9)	0.0229 (8)	−0.0020 (7)	0.0052 (7)	0.0032 (7)
C4A	0.0173 (7)	0.0198 (8)	0.0170 (8)	−0.0005 (6)	0.0012 (6)	0.0014 (6)
C5	0.0233 (8)	0.0227 (9)	0.0193 (8)	0.0011 (7)	0.0098 (6)	0.0007 (7)
C6	0.0338 (10)	0.0236 (9)	0.0233 (9)	−0.0048 (8)	0.0101 (7)	−0.0057 (7)
C7	0.0277 (9)	0.0325 (10)	0.0214 (9)	−0.0114 (8)	0.0015 (7)	0.0010 (8)
C8	0.0200 (8)	0.0298 (10)	0.0287 (9)	−0.0054 (7)	0.0019 (7)	0.0055 (8)
C9	0.0223 (8)	0.0252 (9)	0.0269 (9)	0.0008 (7)	0.0111 (7)	0.0039 (7)
C10	0.0227 (8)	0.0228 (9)	0.0178 (8)	0.0063 (7)	0.0081 (6)	0.0011 (6)
C10A	0.0171 (7)	0.0192 (8)	0.0174 (8)	0.0009 (6)	0.0006 (6)	0.0019 (6)

### Geometric parameters (Å, º)

**Table:**

Br1—C5	1.9234 (17)	C4A—C5	1.478 (2)
Br2—C10	1.9145 (17)	C5—C6	1.327 (2)
C1—C2	1.382 (3)	C6—C7	1.465 (3)
C1—C10A	1.397 (2)	C6—H6	0.9500
C1—H1	0.9500	C7—C8	1.326 (3)
C2—C3	1.381 (3)	C7—H7	0.9500
C2—H2	0.9500	C8—C9	1.467 (3)
C3—C4	1.380 (3)	C8—H8	0.9500
C3—H3	0.9500	C9—C10	1.327 (2)
C4—C4A	1.398 (2)	C9—H9	0.9500
C4—H4	0.9500	C10—C10A	1.484 (2)
C4A—C10A	1.398 (2)		
			
C2—C1—C10A	121.12 (17)	C5—C6—C7	124.91 (17)
C2—C1—H1	119.4	C5—C6—H6	117.5
C10A—C1—H1	119.4	C7—C6—H6	117.5
C3—C2—C1	119.75 (17)	C8—C7—C6	125.79 (16)
C3—C2—H2	120.1	C8—C7—H7	117.1
C1—C2—H2	120.1	C6—C7—H7	117.1
C4—C3—C2	119.95 (17)	C7—C8—C9	125.45 (17)
C4—C3—H3	120.0	C7—C8—H8	117.3
C2—C3—H3	120.0	C9—C8—H8	117.3
C3—C4—C4A	121.04 (17)	C10—C9—C8	125.57 (16)
C3—C4—H4	119.5	C10—C9—H9	117.2
C4A—C4—H4	119.5	C8—C9—H9	117.2
C10A—C4A—C4	119.05 (16)	C9—C10—C10A	127.81 (16)
C10A—C4A—C5	122.07 (15)	C9—C10—Br2	117.29 (13)
C4—C4A—C5	118.88 (15)	C10A—C10—Br2	114.52 (12)
C6—C5—C4A	128.45 (16)	C1—C10A—C4A	119.06 (16)
C6—C5—Br1	117.39 (13)	C1—C10A—C10	118.30 (15)
C4A—C5—Br1	113.91 (12)	C4A—C10A—C10	122.64 (15)
			
C10A—C1—C2—C3	0.6 (3)	C7—C8—C9—C10	−57.1 (3)
C1—C2—C3—C4	0.9 (3)	C8—C9—C10—C10A	−6.8 (3)
C2—C3—C4—C4A	−1.2 (3)	C8—C9—C10—Br2	−179.26 (14)
C3—C4—C4A—C10A	0.0 (2)	C2—C1—C10A—C4A	−1.7 (2)
C3—C4—C4A—C5	179.50 (16)	C2—C1—C10A—C10	177.97 (15)
C10A—C4A—C5—C6	−62.3 (2)	C4—C4A—C10A—C1	1.4 (2)
C4—C4A—C5—C6	118.2 (2)	C5—C4A—C10A—C1	−178.05 (15)
C10A—C4A—C5—Br1	123.72 (14)	C4—C4A—C10A—C10	−178.29 (15)
C4—C4A—C5—Br1	−55.72 (18)	C5—C4A—C10A—C10	2.3 (2)
C4A—C5—C6—C7	2.9 (3)	C9—C10—C10A—C1	−118.0 (2)
Br1—C5—C6—C7	176.62 (14)	Br2—C10—C10A—C1	54.57 (18)
C5—C6—C7—C8	58.2 (3)	C9—C10—C10A—C4A	61.7 (2)
C6—C7—C8—C9	1.3 (3)	Br2—C10—C10A—C4A	−125.75 (14)

## References

[bb1] Allen, F. H. (2002). *Acta Cryst.* B**58**, 380–388. 10.1107/s0108768102003890 12037359

[bb2] Barton, J. W., Henn, D. E., McLaughlan, K. A. & McOmie, J. F. W. (1964). *J. Chem. Soc.* pp. 1622–1625.

[bb3] Bender, C. O., Bengtson, D. L., Dolman, D., Herle, C. E. L. & O’Shea, S. F. (1982). *Can. J. Chem.* **60**, 1942–1952.

[bb4] Bender, C. O., Bengtson, D. L., Dolman, D. & O’Shea, S. F. (1986). *Can. J. Chem.* **64**, 237–245.

[bb5] Bender, C. O., Clyne, D. S. & Dolman, D. (1991). *Can. J. Chem.* **69**, 70–76.

[bb6] Bender, C. O., Dolman, D. & Murthy, G. K. (1988). *Can. J. Chem.* **66**, 1656–1662.

[bb7] Bohshar, M., Maas, G., Heydt, H. & Regitz, M. (1984). *Tetrahedron*, **40**, 5171–5176.

[bb8] Bruker (2008). *APEX2*, *SAINT-Plus* and *SADABS* Bruker AXS Inc., Madison, Wisconsin, USA.

[bb9] Çelik, I., Tutar, A., Akkurt, M., Özcan, Y. & Çakmak, O. (2002). *Acta Cryst.* E**58**, o314–o316.

[bb10] Jones, R., Scheffer, J. R., Trotter, J. & Yap, M. (1994). *Acta Cryst.* B**50**, 597–600.

[bb11] Kidokoro, H., Saito, Y., Sato, M., Ebine, S., Sato, S., Hata, T. & Tamura, C. (1983). *Bull. Chem. Soc. Jpn*, **56**, 1192–1195.

[bb12] Li, W.-K., Chiu, S.-W., Mak, T. C. W. & Huang, N. Z. (1983). *J. Mol. Struct. (THEOCHEM)*, **94**, 285–291.

[bb13] Macrae, C. F., Edgington, P. R., McCabe, P., Pidcock, E., Shields, G. P., Taylor, R., Towler, M. & van de Streek, J. (2006). *J. Appl. Cryst.* **39**, 453–457.

[bb14] Sheldrick, G. M. (2008). *Acta Cryst.* A**64**, 112–122. 10.1107/S0108767307043930 18156677

[bb15] Spek, A. L. (2009). *Acta Cryst.* D**65**, 148–155. 10.1107/S090744490804362X PMC2631630 19171970

[bb16] Westrip, S. P. (2010). *J. Appl. Cryst.* **43**, 920–925.

